# Supplementary figures and images for: Clustering co-abundant genes identifies components of the gut microbiome that are reproducibly associated with colorectal cancer and inflammatory bowel disease
Source: Microbiome. 2019 Aug 1;7:110. doi: 10.1186/s40168-019-0722-6 (PMC6670193; doi:10.1186/s40168-019-0722-6)

**A**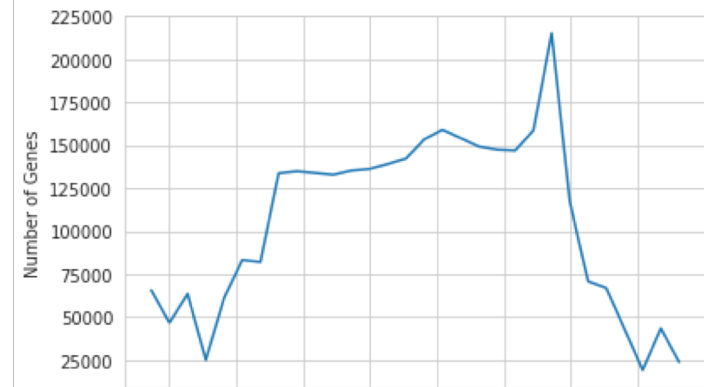**B**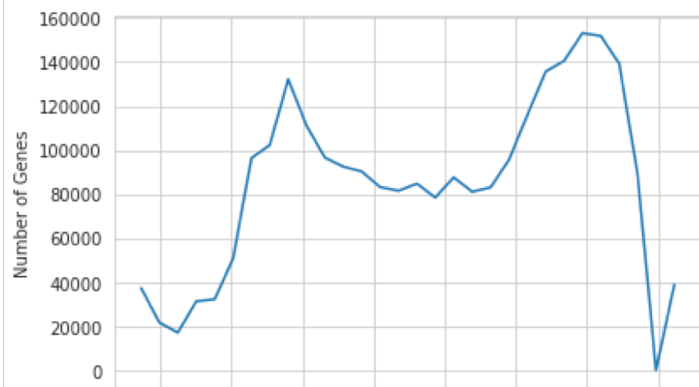**C**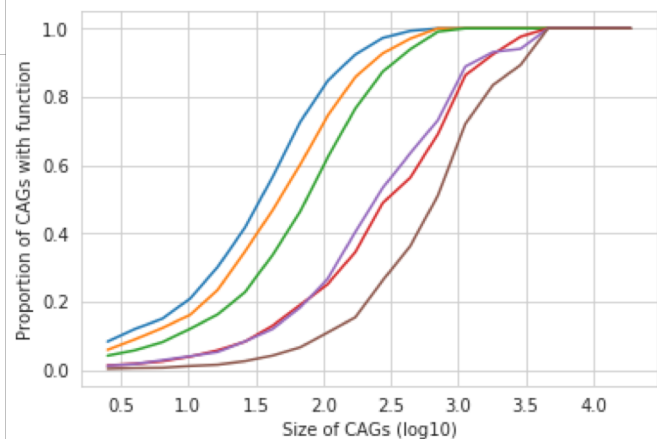**D**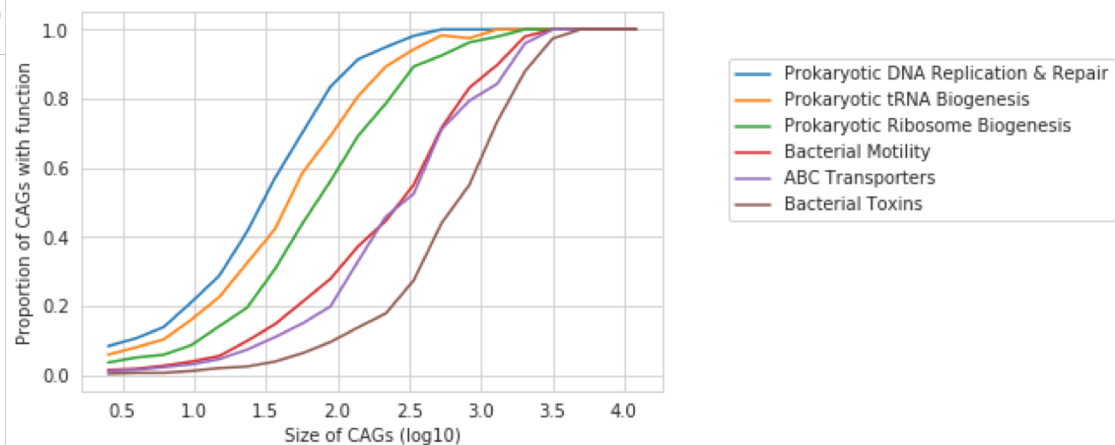

Supplement: Supplementary file 1 — Figure S1. The distribution of CAG size (genes per CAG; A and B) and the functional annotation of genes in CAGs is shown by CAG size (C and D). Each gene can be annotated with a range of biological functions, and the proportion of CAGs of a given size containing at least one functional annotation is shown (C and D). The CAGs generated from the CRC datasets are shown in A and C, while the CAGs generated from the IBD datasets are shown in B and D. The horizontal axis is shared between panels A and C, as well as B and D. (PDF 857 kb) [file 40168_2019_722_MOESM1_ESM.pdf]

CRC-dataset generated CAGs

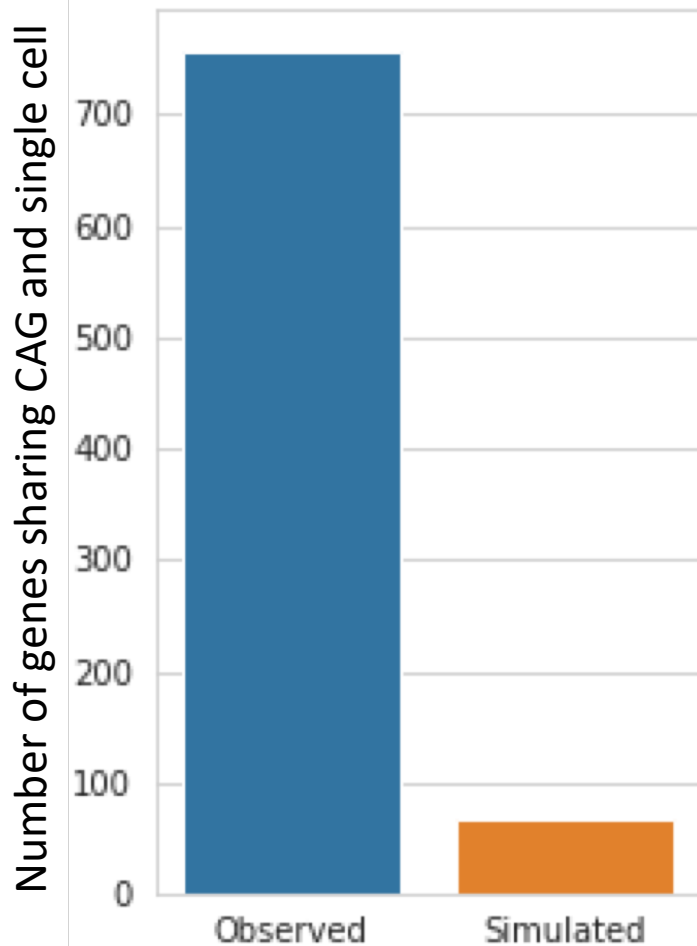

IBD-dataset generated CAGs

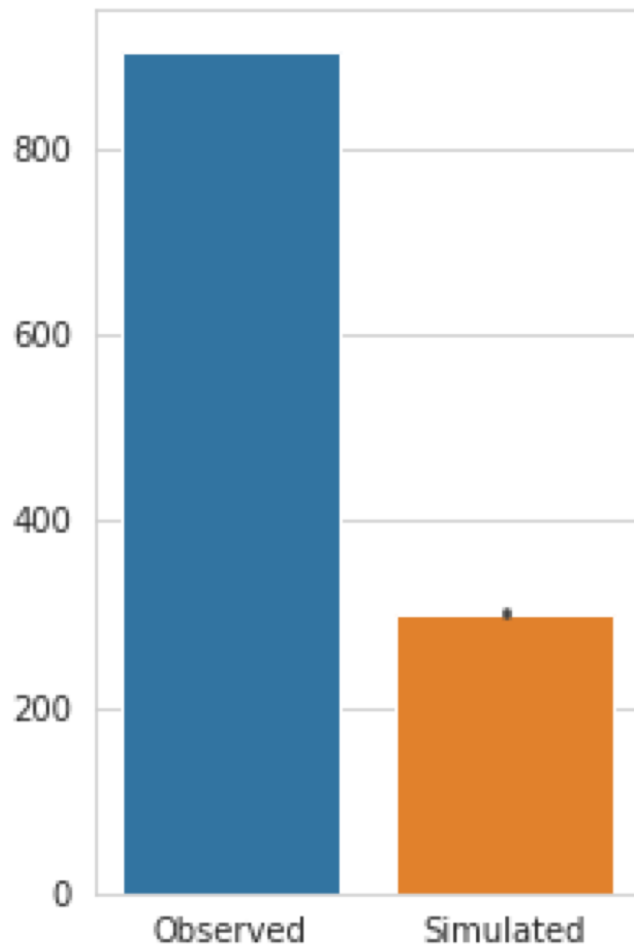

Supplement: Supplementary file 2 — Figure S2. Single-cell microbiome datasets were analyzed using the gene catalogs and CAG groupings from the CRC and IBD datasets. Co-occurrence was measured as the number of genes that were found in the same cell with another gene from the same CAG. Simulations were performed by random permutation, with 1000 replicates. Orange bars show mean and standard deviation. (PDF 288 kb) [file 40168_2019_722_MOESM2_ESM.pdf]

A

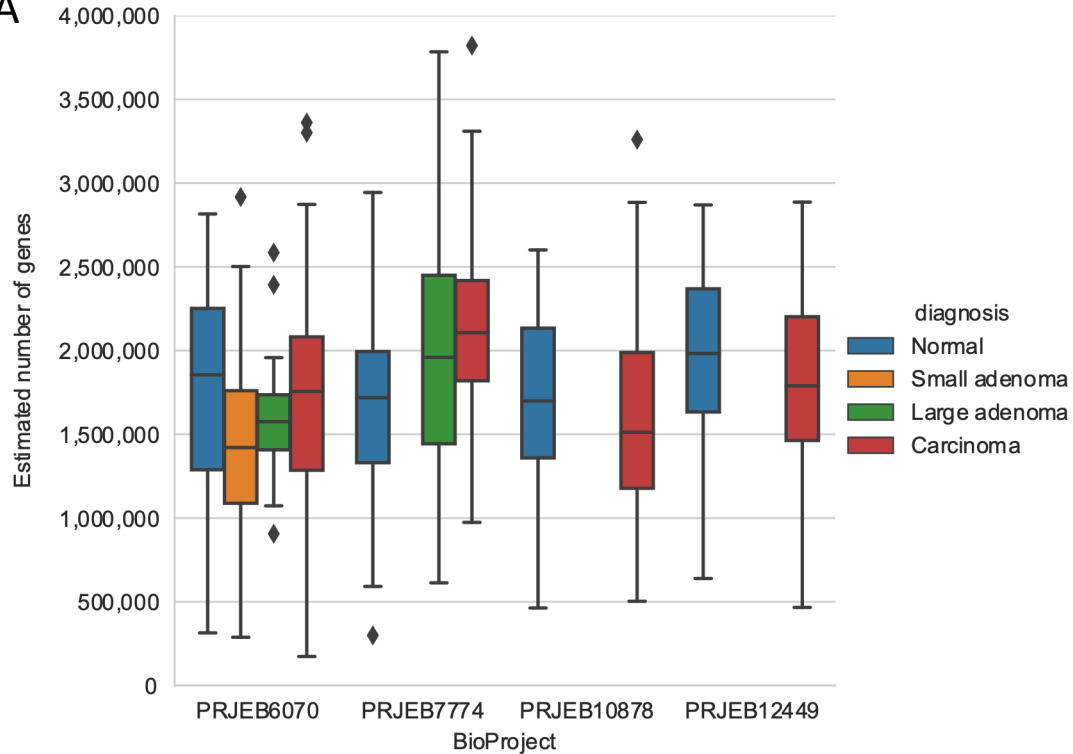

B

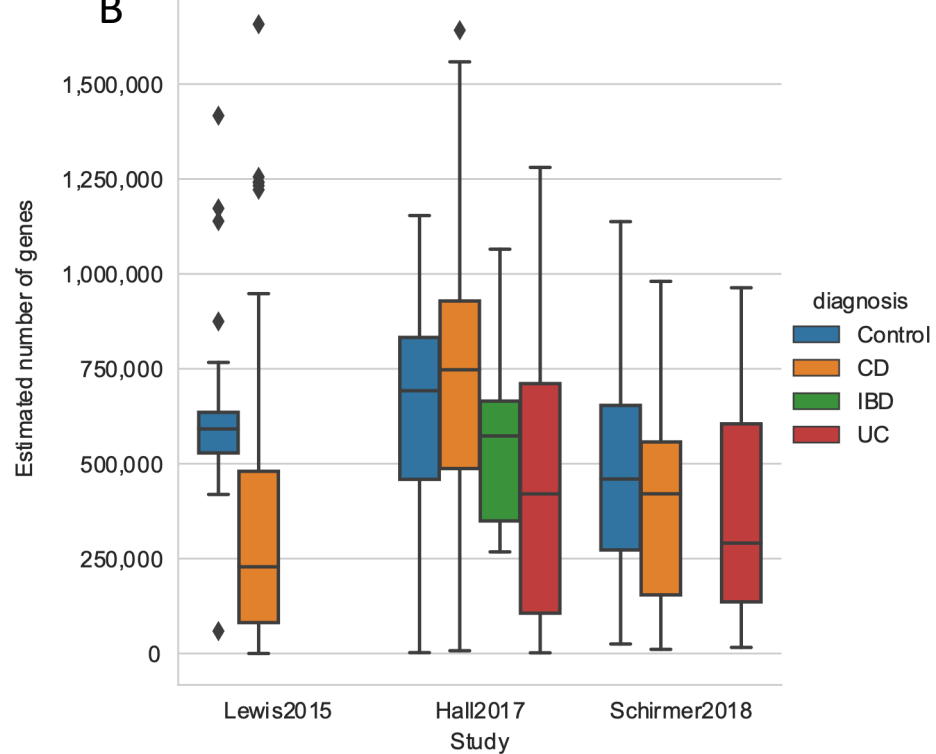

Supplement: Supplementary file 3 — Figure S3. Alpha diversity by diagnosis across cohorts. The number of total genes in each sample was estimated with breakaway for both the CRC (A) and IBD (B) cohorts. (PDF 249 kb) [file 40168_2019_722_MOESM3_ESM.pdf]
